# Sleep walking copepods? *Calanus* diapausing in hypoxic waters adjust their vertical position during winter

**DOI:** 10.1093/plankt/fbab004

**Published:** 2021-02-18

**Authors:** Stein Kaartvedt, Anders Røstad, Josefin Titelman

**Affiliations:** DEPARTMENT OF BIOSCIENCES, UNIVERSITY OF OSLO, PO Box 1066 Blindern, 0316 OSLO, Norway; Red Sea Research Center, KING ABDULLAH UNIVERSITY OF SCIENCE AND TECHNOLOGY, THUWAL 23955-6900, Saudi Arabia; DEPARTMENT OF BIOSCIENCES, UNIVERSITY OF OSLO, PO Box 1066 Blindern, 0316 OSLO, Norway

**Keywords:** *Calanus helgolandicus*, overwintering, dormancy, ocean deoxygenation

## Abstract

While hypoxia is generally associated with negative connotations, some animals may also take advantage of reduced oxygen concentrations. However, the dynamics of such processes for zooplankton are poorly understood. We made continuous acoustic studies of *Calanus helgolandicus* overwintering in hypoxic waters (Oslofjorden, Norway). Their apparent minimum oxygen tolerance was 0.2–0.3 mL O_2_ L^−1^ at 8°C. The copepods adjusted their vertical distribution in concert with the upward progression of hypoxia as oxygen contents declined in the course of winter. The hypoxic overwintering habitat largely excluded potential predators and mortality appeared low in early winter. As the copepod distribution shallowed in phase with declining oxygen contents at depth, mortality increased. In contrast to recent predictions, *C. helgolandicus* had sufficient energy reserves to sustain long-term overwintering. Termination of the overwintering phase in spring was gradual but appeared to accelerate during the development of the spring bloom. Enhanced oceanic deoxygenation with climate change may affect seasonally migrating copepods in unpredictable ways.

## INTRODUCTION

Climate change affects pelagic ecosystems through various mechanisms. For example, higher temperatures cause poleward movements of biogeographic boundaries, also for numerous copepod species (Reygondeau and Beaugrand, 2011; Chivers *et al*., 2017). Oxygen concentrations in the open ocean and coastal waters are declining, and both coastal hypoxia and oceanic deoxygenation are predicted to worsen with increasing global temperatures (Levin and Breitburg, 2015; Breitburg *et al*., 2018). Ocean deoxygenation may cause both losers and winners among plankton (Wishner *et al*., 2020). Some may be excluded from previous habitats, while others may exploit low oxygen waters. Wishner *et al*. (2020) termed organisms being specially adapted to live in oxygen minimum zones for “hypoxiphilic.” Yet, many others may tolerate low oxygen and take advantage of hypoxic habitats on different time scales, e.g. for predator avoidance during either a daily cycle or during diapause (Nolan *et al*., 2019; Wishner *et al*., 2020).

The life cycle of temperate and high latitude copepods often includes a dormant overwintering phase in deep waters. Understanding the dormant life-phase of calanoid copepods has long been an aim of biological oceanography (Pond, 2012). *Calanus* spp. are key copepods in the Northern Atlantic (Conover, 1988). Generally, research has centered on *C. finmarchicus*, but the ongoing northward distribution shift of the congeneric *C. helgolandicus* in response to warming (Fromentin and Planque, 1996; Beaugrand *et al*., 2003), warrants more focus on the less studied *C. helgolandicus* (Bonnet *et al*., 2005).

The dormancy phase of *Calanus* spp. varies among species and regions (Conover, 1988; Kvile *et al*., 2019) and is less understood for *C. helgolandicus* than for its northern congeneric (Bonnet *et al*., 2005; Wilson *et al*., 2015). In some habitats, *C. helgolandicus* seems active in upper waters throughout winter (Conover, 1988), while populations in other locations may overwinter in diapause (Hallberg and Hirche, 1980; Hirche, 1983, 1984). Wilson *et al*., (2015) argued that energy reserves of *C. helgolandicus* would sustain diapause for < 60 days in most of its geographic range, where temperatures exceed 10°C. They reasoned that the potential inability to diapause for longer periods might largely restrict *C. helgolandicus* to continental shelf regions (Wilson *et al*., 2015).

*Calanus helgolandicus* is the prevailing *Calanus*-species in the Oslofjord, as established with both morphological and molecular methods (Beyer *et al*., 1967; Bagøien *et al*., 2000; Bucklin *et al*., 1999). The deepwater of the inner fjord often becomes hypoxic. Bagøien *et al*. (2000) studied the seasonal migration of *Calanus* spp. in the fjord but did not sample in the inner, hypoxic part. We are unaware of studies addressing the tolerance of *C. helgolandicus* to hypoxia. However, *Calanus euxinus* in the Black Sea enters diapause and aggregates near the lower boundary of the oxygen zone (Vinogradov *et al*., 1992; Sakinan and Gücü, 2017). This species is closely related to—and originally was classified as—*C. helgolandicus* (Unal *et al*., 2006). Similarly, Osgood and Checkley (1997a, b) found high concentrations of resting *Calanus pacificus* just above oxygen deficient bottom waters in Santa Barbara Basin. There, the copepods adjusted their distribution upwards as oxygen concentrations declined, but still inhabited waters with very low oxygen levels (Osgood and Checkley, 1997a, b).

We made continuous acoustic measurements for ~4 months of *C. helgolandicus* overwintering in hypoxic, waters of the inner Oslofjord. The acoustic target identity was confirmed with intermittent net sampling during the study period. The acoustic observations documented apparent patterns in mortality, adjustments of vertical distribution reflecting limits of tolerance to hypoxia, as well as duration of the overwintering phase. The acoustic data moreover unveiled small-scale vertical patchiness of the overwintering population in the enclosed basin water below sill depth. While the study presents novel data with an unsurpassed temporal and vertical resolution on this particular *Calanus* species, it more generally represents a model of a diapausing copepod population responding to a changing physical and biological environment during the course of dormancy.

## METHODS

### Study site

We studied overwintering *C. helgolandicus* in the 150 m deep Bunnefjorden, the inner branch of the Oslofjord (59.792171°N, 10.726776°E; Fig. 1 in Klevjer and Kaartvedt, 2011). This is a very sheltered location separated from the outer parts of the fjord by a 57 m deep sill. The residence time of the deep waters is about 3–4 years (Gade, 1973) and waters below the sill commonly become hypoxic (Solberg *et al.,* 2015).

### Acoustics

We made acoustic measurements at 200 kHz for 4 months during winter and spring 2007–2008, supplemented by intermittent field campaigns. The virtual absence of other macrofauna in the low oxygen water (e.g. Solberg and Kaartvedt, 2017) facilitated the acoustic target identification and enabled the use of instrument settings that made the echosounder sufficiently sensitive to record the small and weak copepod targets (see below). Mutlu, (2003) and Sakinan and Gücü, (2017) have previously documented the feasibility of using acoustic data at 200 kHz to assess distribution and biomass of dense concentrations of *Calanus* (*euxinus*) entering oxygen minimum zones of the Black Sea. De Leo *et al*., (2018) applied acoustic data (2 mHz ADCP) from cabled observatories at the west coast of Canada in assessing overwintering among *Neocalanus* spp.

Details on methods and procedures are given in Røstad and Kaartvedt (2013); Solberg *et al*. (2015) and Solberg and Kaartvedt (2017) who studied krill and fish in this fjord branch. In short, we deployed a bottom-mounted upward-facing 200 kHz echosounder (Simrad EK60) at 150 m depth from 6 December 2007 to 17 April 2008. The acoustic transceiver (GPT) was mounted in a pressure-proof casing and connected to land with a cable that both provided electricity and transmitted digitized signals to a computer onshore. The echosounder recorded data for the whole winter with a temporal resolution (ping rate) of 1–2 registrations s^−1^, except during equipment failure 14–20 December 2007 and between 28 December −10 January 2008, due to theft of the land-based logging computer.

The acoustic data were plotted in MATLAB. We present a diel echogram covering the whole water column using an acoustic threshold of −80 dB to illustrate how an oxycline acted as a “false bottom” excluding most macrofauna. We subsequently focus on the deeper hypoxic part, using lower thresholds (−90 and −100 dB) better unveiling the weak copepod targets. We present acoustic data on time scales ranging from 24 h to a composite of the whole study period. In comparison of acoustic data with net catches, we made average echograms for months, as in Staby *et al*. (2011). To get an acoustic estimate of *Calanus* total biomass, we for the first part of the study period integrated the total backscatter between 90 m and 149 m depth (1 m above the seafloor) and averaged every hour. After 1 January 2008, there was noise close to the seafloor, due to gas bubbles from the echosounder, and *Calanus* had moved higher in the water column. The integration was, therefore, shifted upwards from 80 to 140 m depth). This upper limit may have missed some *Calanus* at the end of the study period when the copepods stayed shallower but were necessary to avoid the inclusion of stronger targets that appeared at shallower depths. To eliminate noise and any stronger targets than *Calanus*, the upper integration threshold was set to a backscatter of −70 dB, with a lower threshold of −90 dB. The integrated acoustic backscatter is presented as Nautical Area Scattering Coefficient (m^2^nmi^−2^, NASC).

### Field campaigns

We conducted field campaigns in December 2007 and in January, February and April 2008. Vertical profiles of temperature and salinity were measured by CTD (Conductivity, Temperature and Depth) equipped with Niskin bottles. Water samples from 13 depths between the surface and 150 m (bottom) were analyzed for oxygen content using the standard Winkler method (except for April). Vertical profiles of temperature, salinity and oxygen from the entire water column are given in Solberg and Kaartvedt (2017; their Fig. 2). We therefore here only provide data on density and oxygen below 60 m. Daytime vertical net tows for mesozooplankton (except for January) were made over six depth intervals (bottom–100 m, 100–80 m, 80–60 m, 60–40 m, 40–20 m, 20–0 m) using a WP-2 net. Species identification of *Calanus* was made based on microscopically checking the coxapodite curvature of the fifth pair of swimming legs (cf. Sars, 1903; Fleminger and Hulsemann, 1977) on 20 individuals in samples from the bottom to 100 m (December), 100–80 (February) and 80–60 (April). Only *C. helgolandicus* occurred in these subsamples (mostly stage CV). We, therefore, assigned *Calanus* sp. in the remaining samples to *C. helgolandicus*.

## RESULTS

### Diel echogram at threshold −80 dB

**Fig. 1:**
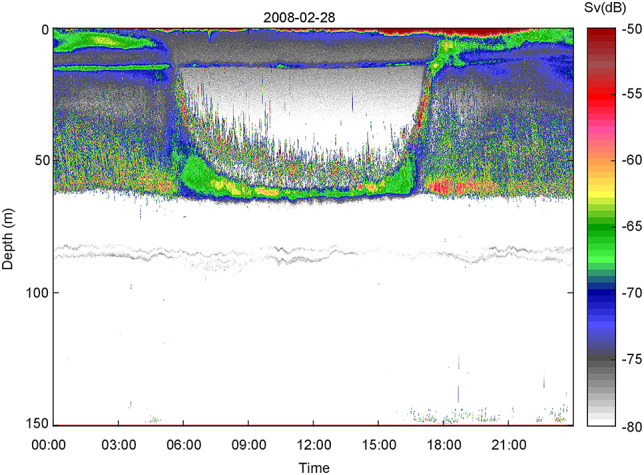
Diel echogram (28 February) encompassing the whole 150 m water column in inner Oslofjorden (Bunnefjorden). At this acoustic threshold of −80 dB, backscatter is largely restricted to the upper ~70 m, only two thin layers of very weak backscatter (~80–90 m) were recorded in the lower part of the water column. Near-bottom echoes are noise. Color scale refers to volume backscatter *(S*v).

A diel echogram (February) illustrates how hypoxic waters (see below) excluded much of the macrofauna (Fig. 1). Echoes of vertically migrating krill and fish prevailed in the upper 60 m. There was a sharp boundary toward waters below, which were largely devoid of backscatter at a −80 dB threshold, apart from some weak backscatter at ~ 80–100 m (Fig. 1). There was no diel pattern in the hypoxic waters.

### Hydrography, net tows and low-threshold acoustic (−90 dB)

**Fig. 2:**
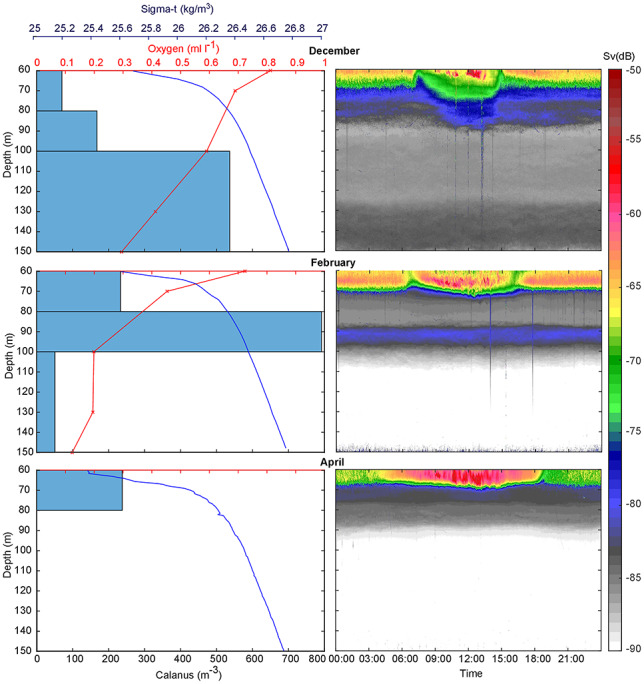
Net catches of *Calanus* 13 December, 12 February and 18 April with red vertical oxygen profiles (13 Dec and 12 Feb) and blue density profiles superimposed (left). Average monthly backscatter for the lower, hypoxic part of the water column is given at an acoustic threshold of −90 dB (right). The strong echoes above ~70 m represent the lower part of the macrofauna inhabiting the more oxygenated part of the water column. Color scale refers to volume backscatter *(S*v).

The temperature was about 8°C and salinity 33 in the deeper part of the water column all winter, with only slight changes with depth in the basin water (data presented in Solberg and Kaartvedt, 2017). The density increased slightly toward the bottom, most sharply above about 80 m. Profiles were very similar throughout the study period, though bottom waters appeared slightly less dense at the end of the study (Fig. 2). Oxygen concentrations were < 0.8 mL O_2_ L^−1^ below 60 m and declined both with depth and time (Fig. 2). Oxygen concentrations in waters just above the bottom were about 0.3 mL O_2_ L^−1^ in December and 0.1 mL O_2_ L^−1^ in February. At 130 m concentrations declined from 0.4 to 0.2 mL O_2_L^−1^ in the course of that period and a corresponding declining trend occurred throughout the basin water.

*C. helgolandicus* CV made up nearly 90% in terms of numbers of the zooplankton captured in the hypoxic waters below 60 m, with the small copepods *Oithona* and *Oncea* dominating the remainder. In December and early winter, abundances were high in deeper parts of the water column with an average of ~500 individuals m^−3^ in the 50 m depth interval from100 m to the bottom. Abundance declined with shoaling depth (Fig. 2). In February, the copepods stayed shallower, with peak concentrations of ~800 copepods m^−3^ at 100–80 m, while concentrations below had decreased markedly. The integrated abundance of *Calanus* in net catches declined from 31 540 individuals m^−2^ in December to 23 050 individuals m^−2^ in mid-February. In April, the copepods had virtually disappeared below 80 m. Stage CVI females now constituted about 50% of the *Calanus*-population and dominated in catches above 60 m (not shown).

The low-threshold acoustic records of the weak targets in the hypoxic waters agreed well with the distribution of *Calanus* in the net samples (Fig. 2). The higher vertical resolution in the acoustic data (averages for whole months) indicates that the majority of *Calanus* captured at 150–100 m in December stems from the lower half of this depth interval. In February, the weak acoustic record in the deepest part of the water column and the marked acoustic maximum at 80–90 m mirrored the distribution of *Calanus* in the net catches. In April, neither catches nor acoustic records documented much *Calanus* below 80 m. There then was a slight discrepancy with somewhat deeper acoustic records compared with the net tows. The acoustic data were averaged for the April records, while the net tows were taken at the end of the study period (Fig. 2).

### Continuous long-term acoustics records in hypoxic waters

**Fig. 3:**
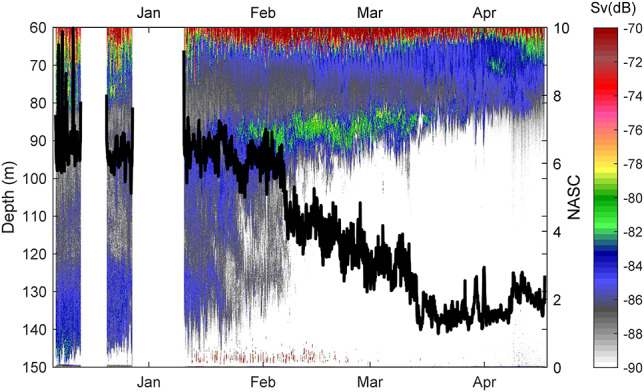
Echogram for waters below 60 m spanning the whole study period. The superimposed black line presents the integrated backscatter (NASC; right axis) as ascribed to *Calanus*. The acoustic threshold is −90 dB. Color scale refers to volume backscatter *(S*v).

At the start of winter, backscatter below the strong echoes of krill and fish in the in the more oxygenated part of the water column increased with depth, apart for a narrow zone just above the bottom, which was devoid of backscatter (Fig. 3). From mid-January, a band of stronger echoes appeared at ~90 m. This band developed into a progressively stronger maximum in February and had disappeared by mid-March. Parallel to the strengthening of this thin layer, echoes became weaker below, until virtually disappearing at ~10 February. Waters below 80 m were devoid of backscatter toward the end of the registration period.

Abundance, as proxied by integrated backscatter (NASC) in the hypoxic waters, initially remained stable with time. There was a sudden decline from early February, with an apparent subsequent accelerated decline from early/mid-March (Fig. 3).

### Patchiness/vertical stratification

**Fig. 4:**
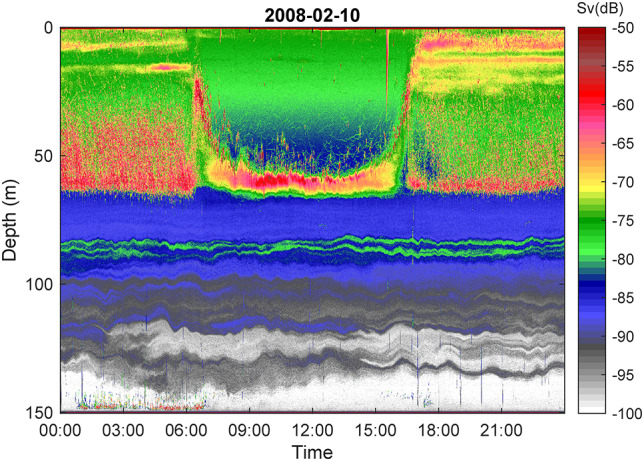
Diel echogram (10 February) at an acoustic threshold of −100 dB, revealing conspicuous vertical layers of backscatter in the lower, hypoxic part of the water column. Near-bottom echoes are noise. Color scale refers to volume backscatter *(S*v).

Resolving the deep acoustic data on a diel scale revealed repeated dense layers alternating with depth intervals without backscatter in the hypoxic water (Fig. 4). The vertical positions of the thin acoustic layers varied somewhat on short-time scales, but not always in synchrony (Fig. 4).

## DISCUSSION

We have presented results on seasonally migrating copepods, applying an echosounder cabled to shore providing data of supreme temporal and vertical resolution. As the backscatter followed a seasonal development similar to that of *C. helgolandicus* in net samples and because there were no alternative acoustic targets in the hypoxic waters, we used the continuous long-termed acoustic records to assess the abundance and vertical distribution throughout winter.

While *Calanus finmarchicus* generally diapause, *C. helgolandicus* appears more variable (Bonnet *et al*., 2005). The *Calanus* overwintering strategy depends on lipid energy storage. Wilson *et al*. (2015) argued that the period of dormancy would be less than 60 days in *C. helgolanidcus* at temperatures comparable to those in our study. However, our direct observations revealed a much longer overwintering period. In Oslofjorden *C. helgolandicus* initiates overwintering and descends during September (Bagøien *et al*., 2000). Regardless of any inter-annual variations, the overwintering population was well established at depth when we initiated acoustic sampling in early December. An accelerated decline in the acoustic backscatter ascribed to *Calanus* during March (cf. Fig. 3) was likely influenced by the initiation of the seasonal ascent associated with the termination of overwintering. This timing matched with the development of the spring bloom (Røstad and Kaartvedt, 2013). While most individuals had terminated overwintering by April, some *C. helgolandicus* remained at depth after the bloom. Similar, and probably state-dependent, long stretched periods of diapause termination over several months occur in other, better oxygenated parts of Oslofjorden (Bagøien *et al*., 2000).

Hypoxia tolerant zooplankton living near their physiological limits may respond to very slight changes in oxygen (Wishner *et al*., 2018). As oxygen concentrations declined at depth during winter, the overwintering *C. helgolandicus* adjusted their vertical distribution upwards (Figs 2 and 3). From the vertical relocation relative to the ambient oxygen concentrations, we derive that their lower tolerance is between 0.2 and 0.3 mL O_2_ L^−1^ at 8°C. Such values compare well with those at which *Calanus pacificus* may diapause, also occurring in waters of 0.2–0.3 mL O_2_ L^−1^ (Alldredge *et al*., 1984; Osgood and Checkley, (1997a, b). Also in other settings, zooplankton abundance and biomass appear to decrease sharply when oxygen levels fall below 0.2 mL O_2_ L^−1^ (Longhurst, 1967; Böttger-Schnack, 1996; Ekau *et al*., 2010). There was no change in water density explaining the shallowing distribution. The enclosed basin water was resident throughout winter as evidenced by the continuous decline in oxygen contents and without signs of intrusion of denser waters. However, we cannot exclude the passive upward movement of animals with differences in lipid composition. Lipids aid in buoyancy (Pond, 2012), become sequestrated during winter (Jónasdóttir *et al*., 2019), yet overwintering copepods may be slightly positive buoyant (Visser and Jónasdóttir, 1999).

By overwintering in hypoxic waters, the copepods were partly, but not totally protected from predators. The net samples from 13 December and 12 February suggest some (~30%) reduction in surface integrated abundance in the course of that 2 month period. While the nets only represent two sampling dates without replicates, the integrated backscatter ascribed to *Calanus* provides a continuous picture. Backscatter remained stable until suddenly higher rates of decline appeared from mid-February, concurring with the formation of a dense copepod layer at 80–100 m (Figs 2 and 3).

Some invertebrate predators like chaetognaths can tolerate low oxygen values and may co-occur with copepods in low oxygen waters (Sakinan and Gücü, 2017), but none were captured below 100 m during the current study (Røstad and Kaartvedt, 2013). In this fjord branch fish and krill generally avoid the lower part of the water column when oxygen deficit (e.g. Fig. 1; Kaartvedt *et al*., 2009; Solberg *et al*., 2015). However, planktivorous fish (sprat; *Sprattus sprattus*) made occasional short excursions into the low oxygen water, apparently foraging on the overwintering *Calanus* during this winter (Solberg and Kaartvedt, 2017). In early winter the bulk of *Calanus* inhabited near-bottom waters (Fig. 2) and was out of reach for such short-range and short-time forays, but sprat dived into the shallowing *Calanus*-layer later in winter (Solberg and Kaartvedt, 2017). *Calanus* would become more accessible to the planktivorous fish when moving upwards and concentrating closer to the oxycline that defines the lower depth of the main sprat distribution. *Calanus* indeed dominated the prey in sprat stomachs this particular winter (Solberg and Kaartvedt, 2017). While we cannot exclude other causes of mortality (cf. Daase *et al*., 2014) predation appears to be the most plausible explanation for the enhanced decline in *Calanus* numbers in mid-winter when vertical distribution became progressively shallower. Advection is an unlikely explanation for the observed population decline, as the water properties attested to an enclosed water mass below the sill depth of Bunnefjorden throughout the study.

Similarly to our interpretation, studies elsewhere have shown that hypoxia can lead to a decoupling of predator–prey interactions (Taylor and Rand, 2003), with the spatial extent of the suitable habitat for fish declining during hypoxic events, while zooplankton may find refuge from predation. Thus, hypoxic conditions may reduce the predation risk of zooplankton from pelagic fish (Taylor and Rand, 2003), although examples of fish exploiting high zooplankton concentrations in hypoxic waters also exist. For example, anchovy may make short-range/term excursions into hypoxic waters with concentrated zooplankton prey (Taylor *et al.*, 2007). Alternatively, if enhanced hypoxia would force the copepods even further upwards, low oxygen might instead be detrimental by making the copepods more vulnerable to visual predators, as suggested for other predator/prey relations with expanding oxygen minimum zones (e.g. Netburn and Koslow, 2015). In this way, hypoxia-related to coastal eutrophication and climate change may alter trophic fluxes through food webs in non-linear ways. The effects of long-term climate-driven changes remain hard to predict, also because marine food webs are more complex than simply considering the spatial overlap between plankton and fish as focused on here (Ekau *et al*. 2010).

The backscatter ascribed to overwintering *Calanus* displayed a strikingly stratified vertical structure. We have not resolved the role of passive versus active accumulation. Pond (2012) argued that achieving neutral buoyancy is essential for copepods that overwinter in diapause since active swimming will both attract predators, and deplete metabolic energy reserves. Zooplankton may accumulate passively in response to density gradients in the environment (Tiselius *et al*., 1994). Some slight apparent vertical variations on short-time scales (Fig. 4) probably reflected physical displacements of the water rather than individuals actively relocating. However, layers and aggregations may also form actively in response to chemical (e.g. Heuschele and Selander, 2014) or physical conditions, invoked both directly by the environment per se (Buskey *et al*., 1995) or indirectly via for example cues emitted from predators or conspecifics (Heuschele and Selander, 2014; Leising and Yen, 1997). Copepods have a whole suite of sensory abilities and can detect and process at least chemical, hydrodynamic and light signals and alter their behavior and distribution in response, so sensory and behavioral mechanisms for maintaining such layers are in place.

## CONCLUSION

*C. helgolandicus* in the Oslofjord sustained long-term overwintering in hypoxic waters. Their apparent minimum oxygen tolerance was 0.2–0.3 mL O_2_ L^−1^ at 8°C. The copepods adjusted their vertical distribution in concert with the upward progression of hypoxia as oxygen contents declined in the course of winter. Such apparent active shifts in vertical distribution during diapause have implications not only for our understanding of altered distribution patterns in response to future climate scenarios but also for predictions of population mortality during winter.

### DATA ARCHIVING

Data are available from the corresponding author upon request.
